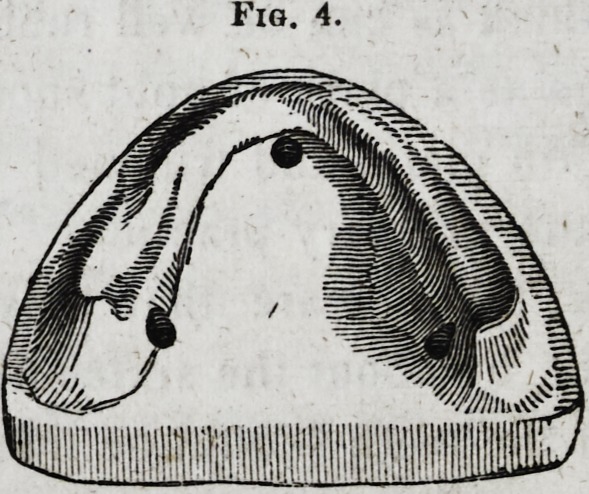# On the Use of Tin as a Base for Artificial Teeth

**Published:** 1850-10

**Authors:** Geo. E. Hawes


### AMERICAN JOURNAL

OF

### DENTAL SCIENCE.

Vol. I. NEW SERIES—OCTOBER, 1850. No. 1.

### ARTICLE I.

### *On the Use of Tin as a Base for Artificial Teeth.*

Dr. C. A. Harris:

*Dear Sir:*—In compliance with your request, made to me at the last session of the American Society of Dental Surgeons, held at Saratoga, I send you the cuts representing the progressive stages and manipulations for casting and gilding whole under sets of artificial teeth with pure tin. I am aware that, in doing this, I am bringing into notice a system of practice which has hitherto been confined to a few only in our profession, and which will not, probably, at first view, meet with general approbation, as it is yet a question, in the theory of some among us, whether tin is admissible in our practice, for constructing dental substitutes.

In the curative treatment of carious teeth, *tin* has been successfully used as a filling; and if it has ever been injuriously affected, it has been owing to the unhealthy secretions of the mouth. But we find that, in the absence of all the human teeth, as in the case of infants and old persons, the mouth and breath are more sweet, and the secretions more healthy, than where they are all or in part remaining. Consequently, with proper attention to cleanliness, pure tin would be much less liable to be injured by that agent, when used as a base for artificial teeth, than as a filling, provided that the work is well adjusted, and does not cause excoriation. Where the mouth is wounded, or unhealed from the recent extraction of teeth, the tin in contact with such parts, (as I notice in my experiments for temporary sets,) becomes rough and corroded by the action of the buccal fluids, which are always acidulated when the mouth is in this condition. To prevent this, when I do not design to gild the work, I use a thin plate of gold under the tin, where it comes in contact with the gum.

The peculiar charm which gold possesses, will always secure for it a preference, with many of our patients, to any of the inferior order of metals, even though they could be furnished with a superior article for real service and comfort, at less expense. Some of our profession also consider it as empirical practice, for no other reason than the *tinkerish* way in which they are constructed, and the cheapness of the material, and fear that if generally adopted, "this our craft is in danger to be set at nought."

But from all that I can learn, from extensive experiment and inquiry, notwithstanding the objections urged against it, I am still of opinion that pure tin gilded, or without gilding upon a thin gold plate, may be used in all cases of whole or fractional under sets of artificial teeth, with more comfort and advantage to the patient, and with less expense and labor to the operator, than is possible with the use of gold alone. The evidence which I have collected in favor of this system of practice, appears to me sufficient to establish the fact that it is no longer matter of inquiry or experiment, whether tin be admitted in our practice as a base for artificial teeth, but that it is a scientific truth, and that every practitioner will, upon examination, find it his duty to recommend it to his patients, as best suited to secure the advantages which they require.

The manner of constructing sets of teeth upon this plan may be varied in different ways, and produce the same results. For fractional sets, it will be necessary to prepare, in the usual way, a thin gold plate, and strengthen that part which comes in contact with the natural teeth which remain in the mouth. When the plate is adjusted, place the wax upon it, and cut it to the right curve and the proper height for the length of the teeth. The teeth are then to be selected and placed round upon the wax, in the proper position for use; but it is not material that they come down to the plate, provided all that remains in view is properly arranged, as all below will be filled with tin when the process is completed. Plaster and sand is now to be put on the outside of the teeth and plate, in the same manner as though they were to be soldered in the usual way. When this is done, the wax may be cut away, the teeth removed from the plaster, and a thin gold back put upon them. In backing them, it will be necessary to bend the platina wires over the gold. The backs may even be soldered to the plate, either by the blow-pipe or soldering iron, thus forming one solid mass of tin, covering the wires, and imitating, as nearly as possible, the form of the alveolar ridge which has been absorbed. When this is done, the plaster may be taken away, and as much tin put upon the front as will restore what has been lost by absorption of gum and alveolar process. When the piece is properly trimmed and burnished, it makes a very strong, and natural set of teeth, in appearance, while the additional weight given to it by the tin keeps it in place better than those made in the ordinary way.

Whole under sets of teeth may be cast of pure tin with great facility, dispensing with all metallic castings and plates of every kind, in the following manner:

After the first cast is procured, which should be made of plaster with a large proportion of sand, fit to it a plate of tin, as thick as can be well rubbed down with a burnisher, and as large as a plate of gold should be. The wax is then put upon the tin plate, and trimmed to the proper curve and height, as in the ordinary practice. Next arrange the teeth upon the wax, taking care that they do not come in contact with the plate, by about the sixteenth of an inch. It is not necessary that the teeth should be lined, but the platina wires should be bent divergingly. The teeth may be broken off with a hammer or ground as most convenient, and arranged in a manner similar to the following cut:

Fig. 1.

Then place a strip of wax around the bottom of the front side of the teeth and plate, concealing all the ragged ends and bad joints. All the wax is now to be carved to represent the natural gums, and to supply the required fullness, See Fig. 2.

Fig. 2.

Care must be taken to select such teeth as have their platina pins low, so that they may remain imbedded in the wax after the carving. When this process is completed, oil the plaster and sand cast, and place the teeth and wax upon it, and pour over them more plaster and sand, so as to cover the whole with a thick mass. After the plaster is thoroughly hardened, the mould may be parted, and the tin plate and all the wax taken away, leaving the teeth secured in the plaster, as Fig. 3 illustrates:

Fig. 3.

Fig. 4.

Apertures must be constructed in the plaster, into which to pour the melted tin, and also for the escape of the air, at points which will not interfere with the subsequent finishing, as marked in Fig. 4.

After washing the platina pins with a flux of muriate of zinc, the two parts of the mould must be securely bound together, and, to insure perfect success, the whole should be slowly heated to the temperature of the melted tin, which it is now ready to receive. Heat pure tin just sufficiently to flow readily, and carefully pour it into the place prepared. When sufficiently cool, remove the plaster, and prepare to polish, first with the file, then with different qualities of sand paper, which executes this work with great facility. Then finish with the same care as for gold. (In cases where gum teeth or blocks are required, the above directions cannot be explicitly followed, as the teeth must then be wholly supported at the base and on the inside.)

The patient should now wear the teeth a few days, so as to become satisfied that they are well adjusted, and that no subsequent alteration will be necessary, as it would deface the work were it done after the gilding.

To prepare the set for gilding, a thorough cleansing is necessary, and unless this is effectually done, the adhesion between the tin and gold will be imperfect, the gold separating from the tin in burnishing, and easily rubbing off. In this process, all grease must be removed by the use of alkaline solutions and afterwards water, then guarding against the moisture of the hand by a glove, perfectly polish the tin with a jeweller's soft brush and prepared chalk. Again rinse in water, to remove all extraneous matter, and immediately place the teeth in the gilding solution. During the process of gilding, the teeth should be removed two or three times and burnished, both to give solidity to the deposit, and to discover imperfections if any exist. When the gold is sufficiently thick, burnish in the usual manner.

I have not fully decided in my own mind, that the constructing of sets of teeth without a thin plate of gold, as first described, is the best method; neither do I as yet feel prepared to speak positively as to the durability of gilding when worn in the mouth, as it is only about one year since I introduced this method into my practice. A few days since, I made a thorough examination of the case, and could not discover any appearance of the gold wearing off.

This is for me sufficient encouragement to pursue the system until time shall render its true and impartial verdict concerning it.

The experience of Dr. C. O. Crosby, of New Haven, corroborates that which I have given, and has the advantage of much longer trial. In answer to some inquiries, he writes to me thus: "Mr. —— has worn his under set, constructed upon tin, for nearly three years, and still perfect. Another patient, Mrs. ——, has a set, which has been in use for two years, still in good condition, but has a silver base or plate, is filled in or loaded with tinner's common soft solder, galvanized with silver, a thin coat, and burnished, and then galvanized with gold, about three coats, and burnished each coating. There is about $2 50 value of gold on each plate. I have about sixty plates made upon this plan. Soft solder plates look dingy unless well galvanized with gold. The galvanizing will stand if there is any gold put on, and they actually require less cleaning, from the fact of the gold being pure. There is no galvanic action when all the other metals are covered. I consider tin alone, without galvanizing, *better* and having *less taste* than *eighteen carat gold*, with copper, silver and gold for solder. I have never found a person that could not wear them."

Dr. Wm. A. Royce, of Newburgh, the pioneer in this practice, has also kindly sent me an account of his experiments, which I forward to you to use as you judge proper, and with many good wishes for the success of your valuable journal, and for the rapid advancement of our important profession,

Allow me to remain,

Truly yours,

GEO. E. HAWES.

## Figures and Tables

**Fig. 1. f1:**
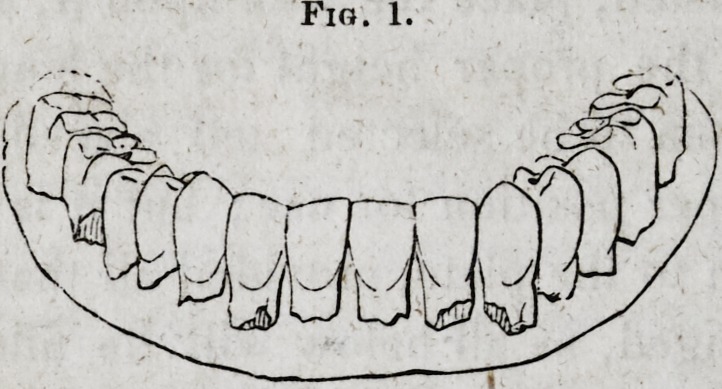


**Fig. 2. f2:**
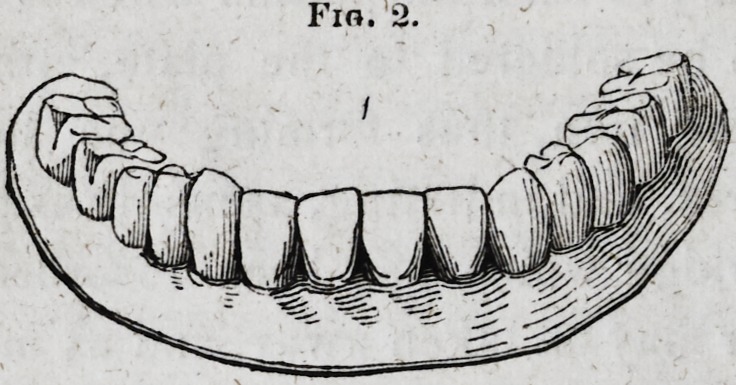


**Fig. 3. f3:**
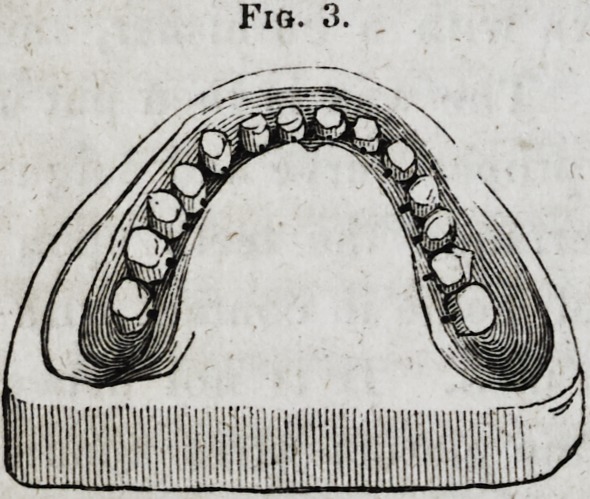


**Fig. 4. f4:**